# More Realistic Face Model Surface Improves Relevance of Pediatric *In-Vitro* Aerosol Studies

**DOI:** 10.1371/journal.pone.0128538

**Published:** 2015-06-19

**Authors:** Israel Amirav, Asaf Halamish, Miguel Gorenberg, Hamza Omar, Michael T. Newhouse

**Affiliations:** 1 Department of Pediatrics, University of Alberta, Edmonton, Alberta, Canada; 2 Ziv Medical Center, Safed, Israel; 3 Nuclear Medicine Department, Ziv Medical Center, Safed, Israel; 4 Firestone Institute for Respiratory Health, St. Joseph’s Hospital, McMaster University, Hamilton, Ontario, Canada; Peking University, CHINA

## Abstract

**Background:**

Various hard face models are commonly used to evaluate the efficiency of aerosol face masks. Softer more realistic “face” surface materials, like skin, deform upon mask application and should provide more relevant *in-vitro* tests. Studies that simultaneously take into consideration many of the factors characteristic of the *in vivo* face are lacking. These include airways, various application forces, comparison of various devices, comparison with a hard-surface model and use of a more representative model face based on large numbers of actual faces.

**Aim:**

To compare mask to “face” seal and aerosol delivery of two pediatric masks using a soft vs. a hard, appropriately representative, pediatric face model under various applied forces.

**Methods:**

Two identical face models and upper airways replicas were constructed, the only difference being the suppleness and compressibility of the surface layer of the “face.” Integrity of the seal and aerosol delivery of two different masks [AeroChamber (AC) and SootherMask (SM)] were compared using a breath simulator, filter collection and realistic applied forces.

**Results:**

The soft “face” significantly increased the delivery efficiency and the sealing characteristics of both masks. Aerosol delivery with the soft “face” was significantly greater for the SM compared to the AC (p< 0.01). No statistically significant difference between the two masks was observed with the hard “face.”

**Conclusions:**

The material and pliability of the model “face” surface has a significant influence on both the seal and delivery efficiency of face masks. This finding should be taken into account during *in-vitro* aerosol studies.

## Introduction

There is an increasing need to better evaluate aerosol devices in ways that more closely mimic clinical use, rather than by simply assessing device performance on the bench [1]. The question of simulating the clinical scenario has become more relevant in young children who use metered dose inhaler (MDI) and valved holding chamber (VHC) with a face mask. The mask has introduced some previously unrecognized variability into the assessment, the two major issues being the mask dead space and the integrity of the seal. It has become evident that it is the seal that is the single most important factor determining the efficiency of aerosol delivery to infants [2].

In order to evaluate optimal seal integrity, various face models have been developed over the past decade. These models have become an essential component for attempting to mimic reality in the laboratory with regard to predicting dead space and seal when the mask is applied to a child’s face. Several face models were developed and reviewed by Mitchell et al [3]. Most of the models described were constructed from rigid materials which were constructed from single silicone layers. These “hard” face models were criticized for being relatively uncompressible (unlike facial tissues in vivo) and insensitive to mask pressure application, with little supporting evidence for their validity as a test-bed that could be used to predict in vivo results. Addition of a layer of soft material to the surface of the “face” is both more costly and complicated than using “hard” silicone. It was suggested that softer materials would deform slightly upon mask application and would allow more relevant in-vitro tests to be performed, particularly with regard to the seal between the mask and “face”. Since the seal is, arguably, the single most important feature of mask performance and one that may be difficult to evaluate in small children, it is important to determine if soft versus hard “face” surface characteristics may affect aerosol delivery to the lower respiratory tract (LRT).

Various features are important in soft face model development, application and validation in order to provide reliable and meaningful information to clinicians treating young children with aerosol medication. These include, mask-application forces, comparison of available devices, comparison with hard face models and use of representative “faces” based on the evaluation of many childrens’ faces. A few pediatric soft face models have been developed [4–7], but none of the previously published studies has taken account of all of these variables.

The purpose of this study was to compare the seal and aerosol delivery of two commonly used pediatric masks by comparing soft vs hard, appropriately representative, pediatric face models using various applied forces between the mask and “face”.

## Methods

### Test Assembly

**Fig 1:**
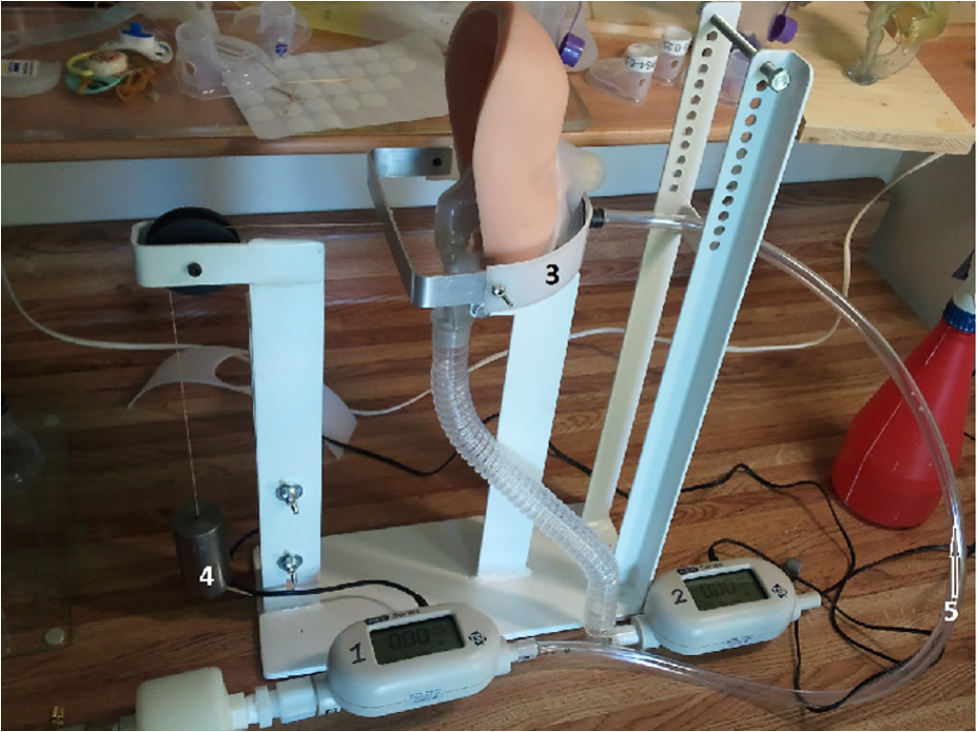
Test Assembly. (#1) Inflow meter represents the baseline ("complete seal") and reflects maximum available inspiratory flow. (#2) Outflow meter input port is connected through flexible tubing to the “trachea”. Its exit port is open to the atmosphere. The airflow measured at this flow meter represents the flow volume through the mask and the model. (#3) Belt holding the mask to the model with various forces (#4).

A test assembly (Fig 1) was designed to hold both the face models and masks and provide various applied forces by means of calibrated weights. These were applied at the back of the test device by means of pulleys and were attached to a light plastic belt anteriorly, that applied the mask to the face. The belt was designed to allow the mask pressure to be influenced solely by the applied forces ranging from 100 to 800 grams.

An air compressor provided a predetermined flow of 7.5 liters / minute through the mask and face model in order to test seal integrity (see below).

### Face masks

**Fig 2:**
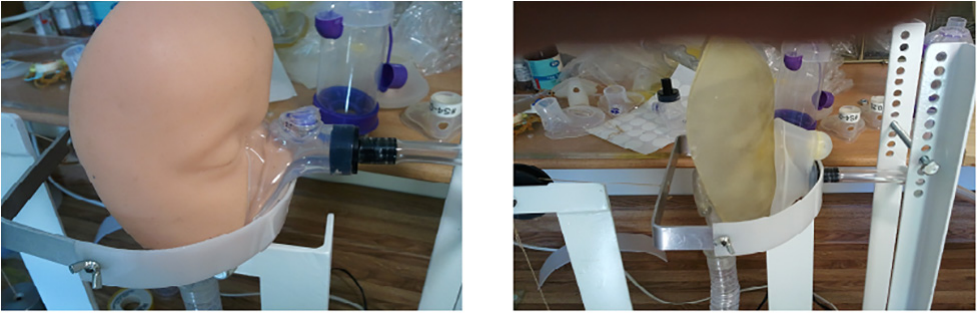
Faces with Masks. (A) Soft face with a SootherMask (B) Hard face with an Aerochamber mask.

Two sets of face masks were used:

1. SootherMask (SM), Medium size (Fig 2A)
2. AeroChamber Plus (AC), Medium size (Fig 2B)

Both masks have exhalation valves mounted in their walls which were sealed for the purpose of this study.

### Face models

The face models were constructed from 3D camera images obtained from 270 children aged 6 months to 3 years that were then divided into 3 “clusters” representing small, medium and large faces. These were obtained in a previous study that provided facial anthropometric data from infants and toddlers [8]. An average medium size 3D face file was transformed to a CAD (Computer Aided Design) file. This file was used to create the inner, firm, supporting layer of the face model using an STL (Stereo Lithographic) file and an Objet Eden 330 3D Printer (Objet Geometries, Rehovot, Israel) to make the insert for a silicone mold. The soft silicone surface of the “face” was created from the mold and was then glued to an STL support (Objet VeroWhitePlus) to obtain a strong durable structure.

Two identical face models were constructed the only difference being the suppleness and compressibility of the outer layer of the “face”. For the outer layer of the “soft face” we used a soft silicone material (Ecoflex 00–30 Smooth-On, Inc. Easton, PA, USA) (Fig 2A). Ecoflex Rubbers are very soft (Shore 00–30 hardness) and provide a surface consisting of very "stretchy/deformable" platinum-catalyzed silicones. This material can be stretched to many times its original size without tearing and rebounds to its original form without distortion. It has a softness and surface that, to the touch, feels similar to the skin and subcutaneous tissue of a baby's face. The second face model’s outer layer was constructed of a harder, more rigid rubber- like silicone material (Objet, Tango, 70–80 Shore A) (Fig 2B). Upper airway replicas for both faces were produced from computerized tomography (CT) scans of the upper respiratory tract (URT) to the level of the tracheal orifice using a CT digital image of a 3 year old child with facial dimensions similar to our model face. This had been previously obtained for medical reasons.

Both face models had exactly the same structure and dimensions and the same “upper airways” extending to the “tracheal” orifice. Both nasal and oral airways were included in the models.

### Seal evaluation

Airflow through the mask was produced by an air compressor that delivered constant flow. Two mass airflow meters (TSI Inc., Shoreview, MN model 4043) were inserted in series–one inflow meter (marked #1 in Fig 1) was placed after the air compressor and before the mask inlet. This meter represents the baseline ("complete seal") and reflects maximum available inspiratory flow. The outflow meter (#2, Fig 1) input port was connected through flexible tubing to the “trachea”. Its exit port was open to the atmosphere. The airflow measured at this flow meter represents the flow volume through the mask and the model. A complete seal would result in identical flow measurements from the two meters. As the mask-“face” seal is broken, leakage increases and the outflow meter will show reduced flow compared to the inflow meter. The percentage leak (inadequate seal; 1/leak volume) can be calculated as the ratio between measurements provided by the two meters. To calibrate the airflow monitors the two meters were connected in series without the masks and face models and the flow velocity measured was compared 5 times. The mean and standard deviation were obtained and they were found to be similar statistically.

The integrity of the seal was then measured during 3 runs for each of the IC and AC masks using either the soft or hard “faces”. Four different forces (100, 200, 400 and 800 grams) were applied for evaluation of the integrity of the seal.

### Aerosol Delivery

#### General

The same test bed as used for the seal evaluation was used for quantification of aerosol delivery with few modifications. Aerosol was delivered to the face models using a valved holding chamber (VHC); Aerochamber (AC) or InspiraChamber (IC) connected to their respective commercial facemasks. A breathing simulator (Harvard Pump, South Natick, MA, USA) was programmed to mimic the breathing pattern and tidal volume of young children. By labeling aerosol with 99mTc and collecting the aerosol at the “tracheal” orifice using absolute filters (Pari GMBH, Munich Germany), we determined the dose delivered and compared it between the two “face” surface textures. Four forces (100, 200, 400 and 800 grams) were applied for aerosol delivery evaluation.

#### Aerosol Generation

Aerosol was generated by a soft mist inhaler that delivers a metered dose of aerosol (Respimat, Boehringer Ingelheim, Ingelheim, Germany). This aerosol generator is powered by a spring-driven piston within a small cylinder. The medication solution reservoir is a plastic cartridge. We found the Respimat system ideal for this study because it was possible to readily radio-label the test solution. The emitted dose, in terms of radioactive counts obtained from placing the absolute filters in a well counter (Capintec Ramsey New Jersey, USA), was shown to be reproducible. For each trial, the MDI canister was filled with 3.0 mL of normal saline radiolabelled with ^99m^technetium (99m Tc). Two ‘puffs‘ from the Respimat were fired one after the other within 10 seconds into the Valved Holding Chamber (VHC) for each run and 3 runs were carried out for each study using either the soft or hard-surfaced “faces”.

#### Breathing Simulator

A computer-operated breathing simulator (Harvard Pump (Harvard Corp., South Natick, MA)) generated a standard waveform at a pre-set “respiratory rate” (RR) and tidal volume (Vt) appropriate for the ‘size’ of ax ‘toddler’. The Vt and RR were chosen based upon the range of actual age—appropriate values [9,10].

#### Aerosol delivery

Radiolabeled aerosol was delivered via the mask during three runs with each of the two models using the 4 applied forces. The breathing simulator ran continuously at the preset variables. For each run, the Respimat mouthpiece was inserted into the back of the VHC and the mask was attached to the surface of the “face” using the pre-determined force. Two successive puffs of aerosol were discharged into the VHC and then “tidal breathing” continued for 30 seconds. This period of time ensured complete evacuation of the aerosol from each VHC.

#### Evaluation of Aerosol Deposition

Aerosol was captured on an absolute non-absorbent filter covering the proximal “tracheal” orifice. Aerosol trapped in this filter represents the mass that would be delivered to the lower respiratory tract (LRT). The drug dose in the filter was quantified by means of a dose calibrator (Capintec, Inc., Ramsey, NJ) and expressed as a percentage of the emitted dose. The dose measured was corrected for decay.

#### Quantification of Emitted Dose

The emitted dose was quantified by measuring the number of microcuries of technetium that exited the outport of the Respimat MDI following two successive puffs into a bacterial filter sealed over the exit port of the device.

#### Data Analysis

Data are presented as mean ± standard deviation for the three runs for each of the models and each of the two face masks. Paired t-tests were carried out to test for differences in filter deposition with the various experimental set-ups.

## Results

### Seal

**Fig 3:**
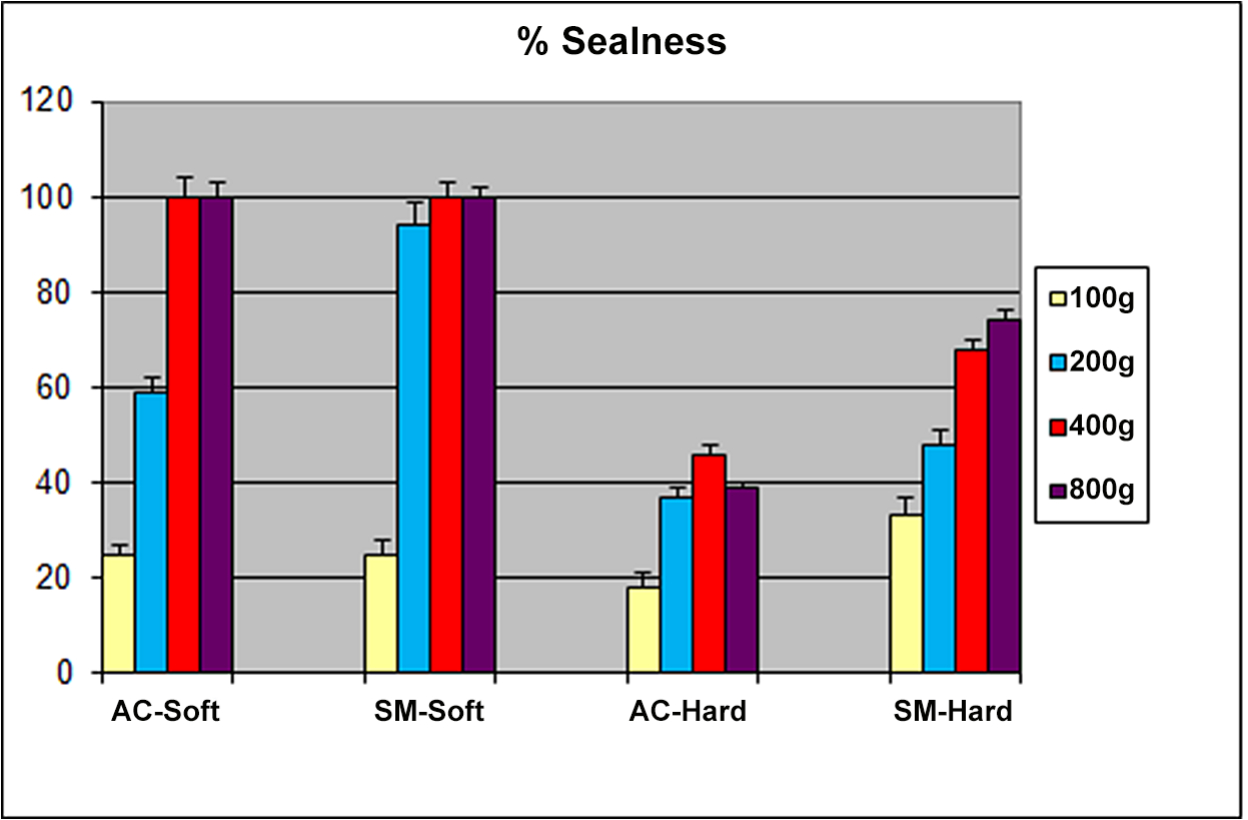
Seal Integrity. Mean (±SD) seal integrity as % for all four applied forces with the two face models (AC-Aerochamber, SM-Soothermask).

The mean seal integrity for all four applied forces with the two face models is summarized in Fig 3.

At the lowest applied force of 100 grams there was no difference in leakage between the “faces” for the two masks (p>0.05). However, with all larger forces (200, 400, 800 gm) seal integrity was significantly greater for the soft compared to the hard “face” (p = 0.02). For example, at 400 grams force, there was 100% seal for the SM and the AC on the soft face model vs 68% (SM) or 46% (AC) on the hard face model. No further improvement in seal was achieved above 400 gm.

When the two masks were compared, the SM achieved almost optimal seal integrity at 200gm whereas the AC required a higher applied mass (400 gm) to obtain the same degree of seal integrity.

### Aerosol delivery

**Fig 4:**
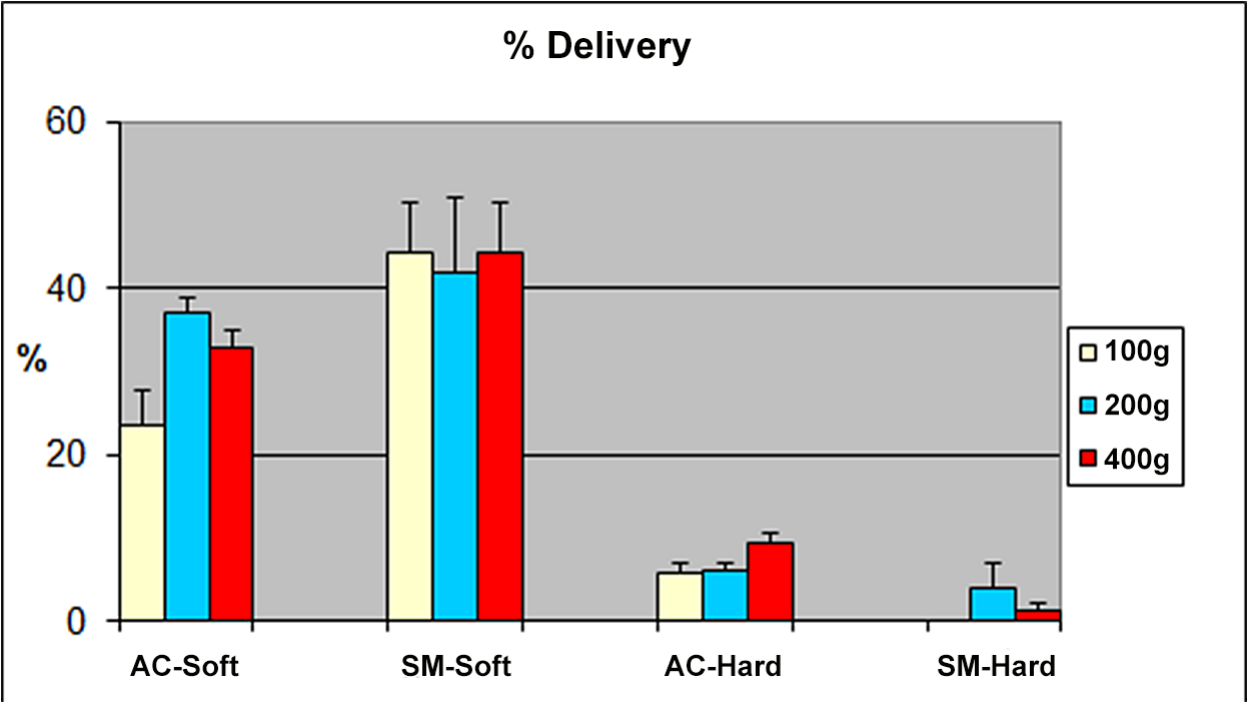
Aerosol Delivery. Mean (±SD) percent aerosol delivery (deposition of 99mTc on the “tracheal” filter) for all applied forces (data for 800 grams not shown) with the two face models.

Mean percent deposition of 99mTc on the “tracheal” filter (i.e. “LRT” deposition) for three forces with the two face models are summarized in Fig 4. As there was no difference for the 800 gm mass for any of the studies, we elected not to show this result here. Data are expressed as percent of the total emitted dose from the Respimat alone.

With all three applied forces (100, 200, 400 gm) delivery of aerosol to the LRT was significantly greater for the soft compared to the hard “face”.

When the two masks were compared, delivery with the soft “face” was significantly greater for the SM compared to the AC with all three applied forc**es**. No statistically significant difference between the two masks was observed with the hard “face” although there was a trend for the AC to provide slightly greater “LRT” delivery.

### Aerosol delivery as a function of the seal obtained

**Fig 5:**
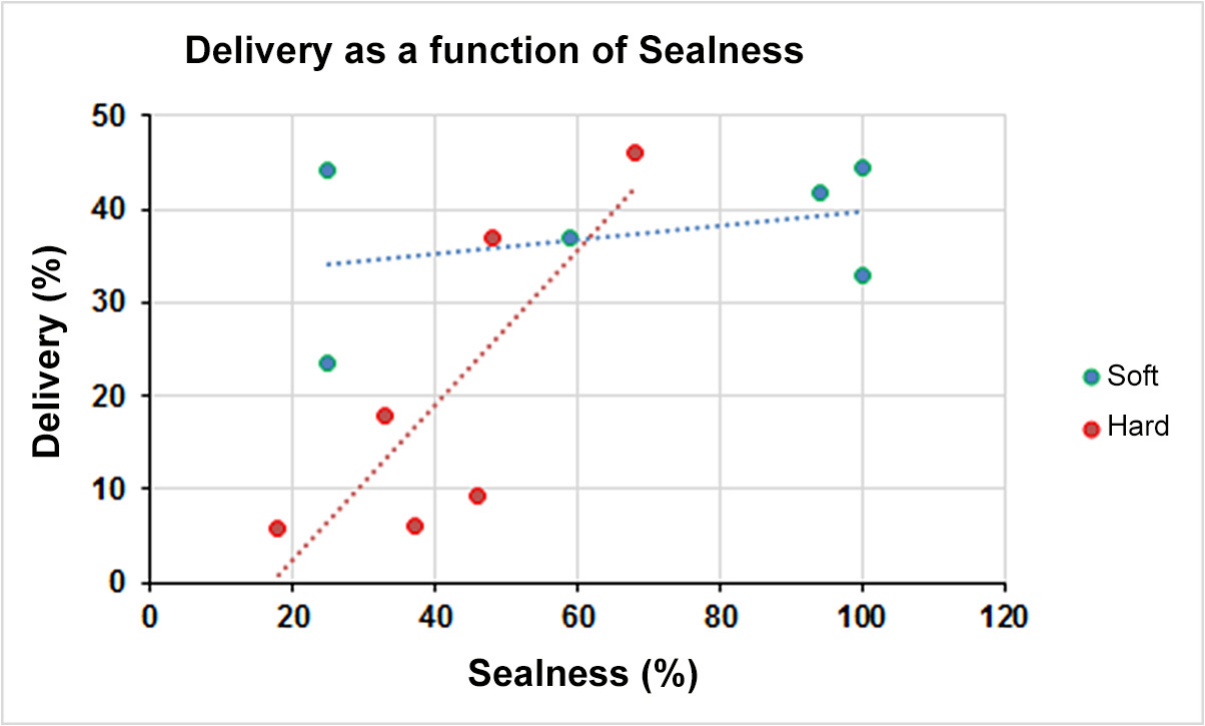
Aerosol Delivery as a Function of Seal. Combined data of aerosol delivery as a function of the seal obtained with the soft (green circles) vs. the hard (red circles) “faces”.

To demonstrate the relationship between “LRT” aerosol delivery and the seal obtained we combined all the deposition data points (for both “faces” and masks) and related it to the combined “sealability” data points (for both “faces” and masks) (Fig 5). Whereas there was a clear relationship between aerosol delivery and he seal in the hard face models, there was no such relationship in the soft face models indicating the importance of “sealability” whenever a hard face is used.

## Discussion

The present study demonstrates that the “pliability” of the “facial” surface plays a major role when evaluating in vitro aerosol delivery to model faces of young children. A recently published article confirmed that many devices used for children have never been tested in them but had only been tested on adults age 18 and older [11]. It is increasingly accepted that children are not simply “small adults” and a device found to be safe and effective in adults may have a very different safety and effectiveness profile when used in a pediatric population, particularly in children under age 3–4 years.

There is thus a great need to apply appropriate methodologies and techniques to accurately represent pediatric scenarios. Aerosol delivery to infants and young children is one of the least researched areas in this regard. A particularly good example are face masks that, until very recently, have simply been size-reduced adult masks and furthermore have not been appropriately tested in infants and toddlers except to establish approximate sizes. In particular, little attention has been paid to the applied force necessary for a seal to be achieved to prevent aerosol leakage and achieve a consistent LRT dose using a soft rather than hard model face surface.

In young children, face masks have been increasingly recognized as arguably the most important link in the chain of aerosol delivery from aerosol generator to the upper respiratory tract. Evaluating face mask performance has lagged well behind other components of aerosol therapy systems such as MDI**s** and VHC**s**. It is clearly important to undertake in vitro studies using faces having surface characteristics similar to those of real children if clinically relevant results are to be obtained. Various features are important in soft-face model development, application and evaluation. More realistic models are needed to provide reliable and meaningful information, from which clinicians can confidently make therapeutic decisions when treating infants and young children with aerosols. These should include appropriate skin-like surfaces, anatomically representative airways, various relevant mask- application forces, comparison of various masks, and the use of representative faces based, as a minimum, on statistically representative real faces. We have been able to find only 4 studies that have used soft face models in pediatric studies. Louca [4] used Plaster-of-Paris to create a “negative” profile of the facial contours of an anatomically correct infant head (Infant Intubation model no. 080001; Laerdal, Stavanger, Norway). No airway was included. Mitchell [5] developed a soft face model (ADAM) including an upper airway which was based on a CT from a 7 month old infant. Carrigy [6] developed a soft face model which was also based on the previous infant CT from the study of Mitchell [5]. An upper airway was included. Xu et al [7] used both a custom-made model of a 4-year-old child’s face (PA Consulting Group, Melbourn, UK) as well as 3D images of two 4 & 5 year old children’s faces to develop their three soft face models. No airway were included. The characteristics of these models are summarized in Table 1. None of previous studies have included all of the components outlined above and were thus an incomplete reflection of childrens’ faces and their upper respiratory tract. The strength of our study stems from the fact, that in contrast to previous studies, all the important features of a face model to evaluate aerosol delivery in young children were included. In particular, the faces used in the present study are more representative as they were produced from anthropometric measurements [8].

**Table 1:** Pediatric studies of soft face models.

1^st^ Author	Year	Age	Face based on*	# of Reference Faces	Airway included	Comparison to Hard face	Comparing various forces	Comparing various devices
Louca	2006	"infant"	Laerdal mannequin head	1	No	No	No	Yes
Mitchell	2010	7m	CT of an infant	1	Yes	No	No	No
Carrigy	2014	7m	CT of an infant	1	Yes	Yes	Yes	No
Xu	2014	4y	"custom-made" (&3D) models of 4&5 year-old children’s faces	1	No	No	No	Yes

*Materials

Louca-Liquid silicone compound (product M-2 liquid silicone compound (product M-2 base and curing agent; Dow Corning Mississauga, Ontario, Canada).

Mitchell-Chemically resistant urethane elastomer.

Carrigy-Shore 05A liquid silicone rubber (LSR-05; Factor II, Inc., Lakeside, AZ), OR- 8 mm layer of Shore 0A polyurethane resin (Hitohada gel; EXSEAL Corporation, Mino City, Japan).

Xu-SkinRite (EnvironMolds, Summit, NJ), a two-part 10 durometer silicone material.

In the present study we have shown that, in a face model study, there is a significant effect of the material from which the mask-“face” interface is constructed, on face mask performance. The soft skin-like surface significantly increased the delivery efficiency and the sealing characteristics of the mask and LRT aerosol delivery efficiency. Previous studies, using hard surfaced ‘faces’, have reported efficiencies for drug delivery with various masks. Since in general the seal integrity was not evaluated in those studies, our finding of a major effect of decreased “sealability” on aerosol delivery in hard face models (Fig 5), calls into question the clinical relevance of those previous findings. Our study has demonstrated that in vitro face models should avoid the use of a hard surface if results obtained *in vitro* are to predict *in vivo* outcomes. A limitation of our study was that we did not attempt to simulate the visco-elasticity and pliability of the skin of a 3 year-old child by means of a specialized instrument to evaluate these properties and future studies could be undertaken to determine if this would further improve the face-mask “sealability”.

The only previous face model study that included a comparison of various masks was that of Louca [4]. The effect of the face material alone was not evaluated. In the present study, we compared two masks and demonstrated that the soft “face” that has surface characteristics more akin to a child’s skin provides greater sealing properties than hard-surfaced “faces” and this can affect the mass of drug delivered to the “LRT” in such models.

Minimizing the force applied to the mask, in order to achieve a seal, is increasingly recognized as an important factor in face mask design. Clearly, the force applied to the mask will influence the seal. However, with small children, application of too great a force in order to achieve a seal may result in discomfort and fear, contributing to rejection of the mask and or crying which has been shown to markedly compromise clinical aerosol delivery. It is thus evident that we need improved masks that achieve a seal with minimal applied force. Xu et al [7] have recently described improved methodology to better evaluate this interesting and important feature of face mask design. In their study, a relatively large force of 1900 grams was applied to the mask in order to achieve a seal. Although this is similar to two other ex vivo studies [5,12], we have shown in the present study that the force necessary to achieve a mask-face seal can be achieved with application of only 200–300 gm, a force which should be less likely to upset the child [13]. Minh et al [14] recently evaluated a new device for measuring flow and force during application of pMDI+VHC with mask in children. This innovative approach uses an electronic device to measure applied force, similar to the one used by Carrigy [6]. Both the electronic and mechanical systems are adequate and both suggest that the forces required to seal the newer masks (e.g. Soothermask and Respironics LiteTouch) to test “faces” are indeed much smaller than previously assumed particularly when using more realistic soft faces. The test pressures applied in our study correlate with those measured in clinical practice. In a recent study with 30 young children using a face mask for aerosol delivery, the mean force (expressed as a weight equivalent) was measured to be 411 gram with a SD of 156 [14]. Thus, the range of forces applied in our study is well within the clinical range.

It must be acknowledged that in contrast to the “face” used in this study, the upper airway model was derived from a single patient. Ethical considerations (e.g., radiation exposure) make it difficult to obtain a large number of “faces” from which appropriate models can be developed. Future development of “idealized” infant and child airway geometry [15] may need to be included in such in-vitro studies.

The significant difference in mask application force and aerosol delivery to the “LRT” between the Soothermask and Aerochamber masks is of interest. The difference was significant both with respect to mask to face seal, and as would be expected, the resulting aerosol delivery to the “LRT”. We postulate that this is due to the SootherMask’s contoured design based on actual facial surface evaluation of a large number of children as well as the 58.3% smaller dead space of the SM (41.7ml) vs AC (71.5ml) [16]. Of particular interest is that this difference would not have been identified had we used only hard-surfaced “faces”. This highlights the importance of using only the more realistically designed soft-surfaced “faces” for evaluation of masks in future *in vitro* studies.
